# Effect of Vermicompost Amendment on the Accumulation and Chemical Forms of Trace Metals in Leafy Vegetables Grown in Contaminated Soils

**DOI:** 10.3390/ijerph18126619

**Published:** 2021-06-19

**Authors:** Yu-Shan Yen, Kuei-San Chen, Hsin-Yi Yang, Hung-Yu Lai

**Affiliations:** 1 Department of Soil and Environmental Sciences, National Chung Hsing University, Taichung 40227, Taiwan; mp850818@gmail.com (Y.-S.Y.); s104039023@smail.nchu.edu.tw (K.-S.C.); 2 Department of Environmental Engineering, National Chung Hsing University, Taichung 40227, Taiwan; ysyieh@gmail.com; 3 Innovation and Development Center of Sustainable Agriculture, National Chung Hsing University, Taichung 40227, Taiwan

**Keywords:** chemical form, lettuce, pak choi, risk assessment, trace metal, vermicompost

## Abstract

(1) Background: Trace metal (TM) contamination of farmland soil in Taiwan occurs because factories dump wastewater into irrigation ditches. Since vermicompost affects the bioavailability of TMs, the objective of this study was to evaluate its effects on the accumulation of growth of TMs in leafy vegetables. (2) Methods: Two TM-contaminated soils and different types of pak choi and lettuce were used and amended with vermicompost. Besides soil properties, the study assessed vermicompost’s influence on the growth, accumulation, and chemical forms of TMs and on the health risks posed by oral intake. (3) Results: Vermicompost could increase the content of soil organic matter, available phosphorus, exchangeable magnesium, and exchangeable potassium, thus promoting the growth of leafy vegetables. The accumulation of four TMs in crops under vermicompost was reduced compared to the control, especially for the concentration of cadmium, which decreased by 60–75%. The vermicompost’s influence on changing the chemical form of TMs depended on the TM concentrations, type of TM, and crop species; moreover, blanching effectively reduced the concentrations of TMs in high-mobility chemical forms. Although vermicompost mostly reduced the amount of cadmium consumed via oral intake, cadmium still posed the highest health risk compared to the other three TMs.

## 1. Introduction

Most farmland contaminated with trace metals (TMs) in Taiwan resulted from illegal discharge wastewater into rice paddy irrigation water. The TMs in irrigation water adsorb onto the sediment at the bottom of irrigation channels and then enter farmland [1]; thus, extensive tracts of farmland are frequently contaminated with TMs, even after remediation, if the sediments are not removed regularly. Of the TMs in question, cadmium (Cd) is nonessential and accumulates easily in plants [2,3]. High concentrations of Cd have a harmful influence on the leafy area, stoma density, chlorophyll content [4,5,6], and photosynthesis rate [5] of plants. High concentrations of copper (Cu) also reduce the photosynthesis rate and the assimilation of nitrogen (N) [7].

Studies have found evidence of the compartmentalization of TMs in cell walls or vacuoles as a detoxification mechanism of plants in response to TM stress [8,9]. The cell wall is mainly composed of cellulous, semi-cellulous, pectin, and protein [10]. These functional groups and proteins in the cell wall can restrict the entrance of TMs into protoplasts [11], which is an important detoxification mechanism for water spinach under Cd stress [12]. The low molecular weight complex of TMs and phytochelatin, which are synthesized from metallothionein and glutathione and then stored in the vacuole, has been observed to reduce the toxicity of TMs [13,14]. Previous studies have revealed that the mobility and toxicity of TMs are determined by their subcellular distribution and chemical form [15]. Wu et al. [16] reported that a higher proportion of Cd was compartmentalized in its inorganic and water-soluble chemical forms in a sensitive wheat variety; however, pectin- and protein-integrated chemical forms were the major chemical forms of TMs in a tolerant wheat variety. Of those chemical forms, inorganic and water-soluble chemical forms of TM have high mobility and toxicity and are easily transported upward to shoots [11,16,17]. Under phosphorus (P) treatment, the proportion of Cd compartmentalized in the pectin- and protein-integrated chemical forms increased, restricting the uptake and upward transfer of Cd in water spinach [18].

Earthworms play an important role in soil properties, the degradation of organic matter (OM), and the transformation of nutrients [19]. The interaction of earthworms and microorganisms can transform different types of OM into vermicompost (VRM). The quality of VRM is determined by the types of organic waste, pH, temperature, and earthworm species [20]. VRM is often applied to treat TM-, pesticide-, and oil-contaminated soils [21,22,23]. Furthermore, VRM application can increase the populations of microorganisms and soil fertility, because of its high OM content and high porosity, cation exchange capacity (CEC), and aeration [24]. Because VRM can form complexes with TMs, the availability of TMs decreases under VRM amendment [25,26,27] and thus, decreases the accumulation of TMs in plants [28,29].

The mobility and availability of soil TMs relate not only to their total concentrations but also strongly to their fractionation [30]. Some studies have reported that total concentrations of TMs decreased after vermicomposting, possibly due to the uptake of TMs by earthworms [31,32,33]; however, the total concentrations of TMs increased in some studies as a result of degraded OM [34,35,36]. Besides total concentrations, the fractionation of TMs is also affected by the vermicomposting process. Song et al. [36] and Yang et al. [37] showed that the availability and mobility of TMs decreased after vermicomposting due to a decrease in the exchangeable fraction of TMs. Similar results were obtained by Santana et al. [38], who revealed that VRM application increased the P and magnesium (Mg) content in jack bean plants (*Canavalia ensiformis* L.), and the concentration of soluble Cu decreased under VRM treatment.

The consumption of TMs from crops and water is the major pathway for the human intake of TMs [39,40], and the total concentrations of TMs in the edible parts of vegetables have been used to assess health risk through vegetable consumption. Because most vegetables are pretreated before cooking, washing and cooking methods affect the chemical forms and thus, the bioaccessibility of TMs [41]. The experimental results of Chen and Lai [3] showed that the bioaccessibility of Cd in rice decreased because the proportion of Cd compartmentalized in the cell walls increased with increases in cooking time and temperature. Relative to raw tissues, boiling and gridding decreased 53–71% and less than 11% of the arsenic (As) in mushrooms, respectively [42].

Different varieties or cultivars of leafy vegetables, with high and low TM accumulation capacities, were selected for a preliminary experiment and then grown in two soils contaminated with essential and nonessential TMs and amended with VRM. The objectives of the study were to assess the effect of VRM amendment on soil properties, the availability of soil TMs, the growth and accumulation of TMs in leafy vegetables and the health risk through oral intake.

## 2. Materials and Methods

Two leafy vegetables (pak choi and lettuce) with high and low TM accumulation capacities, selected previously, were used in this study. The two varieties of pak choi were *Brassica chinensis* L. var. Chinensis (coded as BCC) and *Brassica chinensis* L. cv. Wrinkled leaf (coded as BCW). The two lettuce cultivars were *Lactuca sativa* L. cv. Chinese (coded as LSC) and *Lactuca sativa* L. cv. Sweet (coded as LSS). The experimental results of the previous study showed that the LSC and LSS accumulated 2.1–6.9 and 1.8–3.1 mg kg^−1^ of Cd in the shoots after being grown for 35–49 d; moreover, the BCC and BCW accumulated 2.9–12.7 and 6.6–32.3 mg kg^−1^ of Cd in the shoots after being grown in the same Cd-contaminated soils for 35–49 d [43]. The two varieties of pak choi used in this study had higher accumulating capacity of Cd compared with the two lettuce cultivars. Two species of earthworms (*Eisenia andrei* and *Perionyx excavates*) were grown in waste sawdust from mushroom production. A total of 5.0 kg of waste sawdust were placed in a cuboid box (L 47 cm × W 33 cm × H 18 cm), the water content was adjusted to 70–75% by weighing and adding deionized water (DI water), and then, 0.5 kg of earthworms was added. Rice husk, 50 g every two days, was first administered at 8 d, and the VRM was collected at 60 d.

Surface soils (0–20 cm) were collected from two TM-contaminated sites in central Taiwan, coded as Ti-132 and Ya-400. Soils were air dried, ground, sieved (10, 80, or 100 mesh according to the soil properties to be analyzed), then the basic properties—pH (W_soil_/V_water_ = 1/5) [44], electrical conductivity (EC_w_; W_soil_/V_water_ = 1/5) [45], 2 M KCl extractable N [46], 1 M NH_4_OAc (pH 7.0) extractable cations [47], and available P—were analyzed using the Bray No.1 method and UV spectrometry [48], and the soil OM content [49] was determined. The soils were digested using aqua regia, and the total concentrations of Cu and zinc (Zn) in the filtered (Whatman No. 42 filter paper) digestants were analyzed with inductively coupled plasma atomic emission spectroscopy (ICP-AES; PerkinElmer Avio200, Waltham, MA, USA). Soil wet aggregate stability (WAS) was determined using a wet-sieving device in accordance with the protocol reported by Murer et al. [50].

The VRM was applied according to the total amount of N recommended by the Council of Agriculture of Taiwan and the N content of the VRM. For pak choi and lettuce, the recommended N amounts were 180–240 and 100–120 kg ha^−1^, respectively. The four treatments used in this study were: (1) Ti-132-CK control without applying VRM; (2) Ti-132-VRM, with VRM applied at 53.05 g kg^−1^-soil; (3) Ya-400-CK control without VRM applied; and (4) Ya-400-VRM, with VRM applied at 26.52 g kg^−1^ soil. Soil or soil–VRM mixtures were added to pots (L 11 cm × W 11 cm × H 14 cm), and seeds of leafy vegetables were then sown. The soil moisture content was controlled at 50–70% of water-holding capacity (WHC) by weighing and supporting the DI water. Pot experiments were carried out in a growth chamber (relative humidity 61.2 ± 2.4%, temperature 25.1 ± 1.5 °C) with 14 h of artificial lighting and DI support water administration every two days to maintain the soil moisture content at 50–70% of WHC. Plants were harvested after 60 d of growth, and the relative chlorophyll content of the most extended leaves was assessed using SPAD reading (SPAD-502 Plus, Konica Minolta, Osaka, Japan). Plant tissues were divided into roots and shoots, washed with DI water, and the root lengths and shoot heights were measured. The plant roots were first soaked in a 20 mM Na_2_-EDTA solution for 15 min to remove the adsorbed TMs on the roots’ surfaces [50] and then washed with DI water. The fresh weight of each organ was determined and recorded.

The TMs that had accumulated in the different plant organs were divided into six chemical forms according to Lai [15]: inorganic (F_E_), water soluble (F_H2O_), pectin- and protein-integrated (F_NaCl_), insoluble phosphate complex (F_HAc_), oxalic complex (F_HCl_), and residual (F_R_). Plant tissues were oven-dried at 70 °C for 72 h and the oven-dried plant tissue was ground and digested with nitric acid and perchloric acid (v/v = 4:1) [51], and the concentrations of TMs were determined with ICP-AES (PerkinElmer Avio200, Waltham, MA, USA).

Besides concentration, two indexes were used to assess the accumulation capacity of TM in the two crops and the VRM’s effect. The bioconcentration factor (BCF)—the ratio of TM concentration in the shoot of a plant to that in the soil—was used to examine the accumulation capacity of leafy vegetables. Another index translocation factor (TF)—the ratio of TM concentration in the shoot of a plant to that in the root of the plant—was used to examine the upward transport of TMs under different treatments. The hazard quotient (HQ_v_) was calculated in accordance with Chen et al. [43] to assess the health risk of TM through oral ingestion of vegetables using Equation (1), where C_p_ is the TM concentration (mg kg^−1^) in the edible parts of the four leafy vegetables, MIDVC is the mean individual daily vegetable consumption (kg d^−1^), and TDI_v_ is the tolerable daily intakes through oral ingestion of vegetables (mg kg^−1^-BW d^−1^). Based on the Nutrition and Health Survey report and vegetable calorie counts, the MIDVC in Taiwan during 2013–2016 was 0.133 kg d^−1^ [43]. The TDIs of Cd, Cu, Ni, and Zn set by the European Food Safety Authority (EFSA) [52,53] and the Joint FAO/WHO Expert Committee on Food Additives (JECFA) [54,55] were 0.36, 500, 2.8, and 300 μg kg^−1^-BW d^−1^, respectively.

(1) $${\mathrm{HQ}}_{v}=\frac{\frac{C_{p}\times \mathrm{MIDVC}}{\mathrm{kg}\cdot \mathrm{BW}}}{{\mathrm{TDI}}_{v}}$$

Statistical analysis was performed using the Statistical Package for the Social Sciences (SPSS; Armonk, NY, USA) software. Variance was examined to determine the consequential relationship between soil properties, growth exhibitions, and TM concentrations under the different treatments. One-way analysis of variance (ANOVA) was performed to detect differences in soil properties and plants across treatments. The least significant difference (LSD) test was used to identify significant differences between the means, and a value of *p* < 0.05 denoted statistical significance.

## 3. Results and Discussion

### 3.1. Basic Properties of VRM and Soils

The VRM used had neutral acidity, and its OM content, EC, and carbon/nitrogen (C/N) ratio were 65.52%, 5.39 dS m^−1^, and 16.4, respectively. The total concentrations of N, P, and K were 2.31%, 1.23%, and 1.65%, respectively. Neglectable amounts of TMs in the VRM were determined, and total concentrations of Cd, Cu, Ni, and Zn were not detectable (ND), ND, 0.02, and 8.32 mg kg^−1^, respectively.

The Ti-132 soil was slightly contaminated with Cd and Zn, and the total concentrations were slightly higher than the monitoring standards of the Soil and Groundwater Pollution Remediation Act (SGWPR Act) for farmland of 2.5 mg Cd kg^−1^ and 260 mg Zn kg^−1^, respectively, mandated by the Environmental Protection Agency of Taiwan (EPA Taiwan). The Ti-132-CK had moderate alkalinity and the VRM application decreased the soil pH from 8.2–8.6 to 8.0–8.3; however, the VRM amendment had no significant effect on the soil’s EC, which was recorded at levels of 0.10–0.16 dS m^−1^ (Table 1). Because of the VRM’s high OM content, the VRM amendment also significantly raised the soil OM content compared with CK. For BCC, BCW, and LSS amended with VRM, there were 1–2% increases in the soil OM relative to CK (*p* < 0.05). The concentrations of available P and exchangeable K of CK were approximately 20 and 142 mg kg^−1^, respectively, and these concentrations significantly increased 2.3–5.0 times under VRM treatment (*p* < 0.05). VRM amendment also significantly increased the concentration of exchangeable Mg compared with CK (*p* < 0.05).

The Ya-400 soil was more seriously contaminated with Cd and Ni than the Ti-132 soil, and the total concentrations exceeded the control and monitoring standards for farmland; 5 mg Cd kg^−1^ and 130 mg Ni kg^−1^, respectively, for the SGWPR Act as mandated by the EPA Taiwan. The texture of Ya-400 was silty loam, with a neutral soil pH of 7.1–7.2 and a normal EC of 0.03–0.05 dS m^−1^ (Table 1). The VRM treatment significantly increased the soil pH and EC to levels of 7.2–7.7 and 0.07–0.10 dS m^−1^, respectively (Table 2). Similar to the result for the Ti-132 soil, the soil OM content also significantly increased from 1.3–1.6% (CK) to 2.0–3.1% (VRM). The concentration of the available P content of the Ya-400 soil under the CK treatment was 4.8–9.6 mg kg^−1^, and the VRM treatment significantly increased this value to 24–55 mg kg^−1^ (*p* < 0.05). Similar to the result for the Ti-132 soil and relative to CK, the concentrations of exchangeable Mg and exchangeable K also significantly increased 1.1 to 2.8 times under the VRM treatment (*p* < 0.05).

In addition to the total TM concentrations, the 0.01 M CaCl_2_ extractable TM concentration in the two contaminated soils was also analyzed to assess the VRM’s influence on the availability of TM (detailed data not shown). In general, VRM application decreased the availability of Cd in both the contaminated soils. Most of the 0.01 M CaCl_2_ extractable Cd concentration decreased 17–67% compared with CK, except for BCC; however, no identical result was found for Cu, Ni, or Zn. The CaCl_2_ extractable Cd concentrations of CK for the Ti-132 and Ya-400 soils were 0.03–0.10 and 0.19–0.36 mg kg^−1^, respectively. Under VRM treatment, the CaCl_2_ extractable Cd concentration decreased to levels of 0.03–0.07 and 0.09–0.19 mg kg^−1^, respectively.

### 3.2. Growth Exhibitions

**Figure 1:**
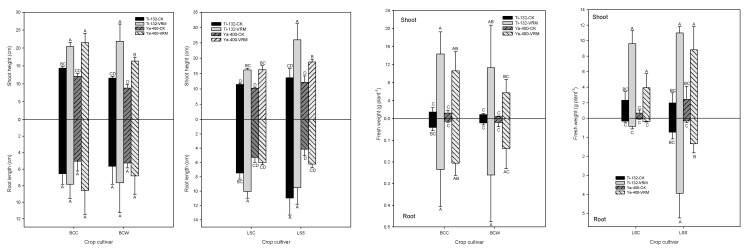
Effects of different treatments on the root length, shoot height, and fresh weight of pak choi and lettuce. The meaning of each code is the same as Table 1. Different letters indicate significant differences across treatments as determined by one-way ANOVA analysis (LSD test, *p* < 0.05, *n* = 3).

**Figure 2:**
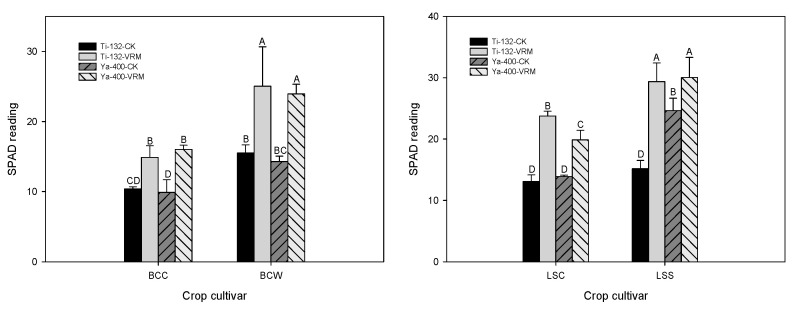
Effects of different treatments on the SPAD readings for pak choi and lettuce. The meaning of each code is the same as Table 1. Different letters indicate significant differences across treatments as determined by one-way ANOVA analysis (LSD test, *p* < 0.05, *n* = 3).

Relative to CK, VRM application generally increased the root length (Figure 1); however, only LSC was statistically significant (*p* < 0.05). The shoot heights of the Ti-132-CK and Ya-400-CK soils ranged from 8 to 15 cm, and the VRM treatment significantly increased the shoot height of the lettuce and pak choi (*p* < 0.05) (Figure 1). There was a 30–50% increase in shoot height under VRM amendment compared with CK. A similar result was found for the fresh weight of the two crops; the VRM amendment significantly increased the fresh weight of the roots compared with CK (Figure 1). The fresh weights of the shoots of lettuce and pak choi grown in CK were 0.4–2.5 g plant^−1^, and the VRM had significantly increased the fresh weight of the shoots of the two crops to levels of 3.9–14.4 g plant^−1^. The SPAD readings for the four crops grown in CK were at levels of 10–15, with a 30–45% increase under VRM treatment (Figure 2). The SPAD readings for the VRM treatment were 13–25 and 20–30 for pak choi and lettuce, respectively.

The concentrations of OM, available P, exchangeable Mg, and exchangeable K of the two soils increased significantly under the VRM treatment compared with CK (Table 1 and Table 2), and these increases could provide the nutrients needed for the growth of lettuce and pak choi. TM stress resulted in chlorosis of the leaves and decreased the metabolism rate and enzymatic activity of plants [56]; however, the experimental results for this study revealed that the VRM amendment was able to alleviate those negative influences on the growth of lettuce and pak choi. In general, the root length, shoot height, fresh weight, and SPAD readings significantly increased under VRM treatment compared with CK. Similar results were reported by previous studies [57,58], which revealed that VRM application can improve soil quality and promote plant growth.

### 3.3. Accumulation of TMs

**Figure 3:**
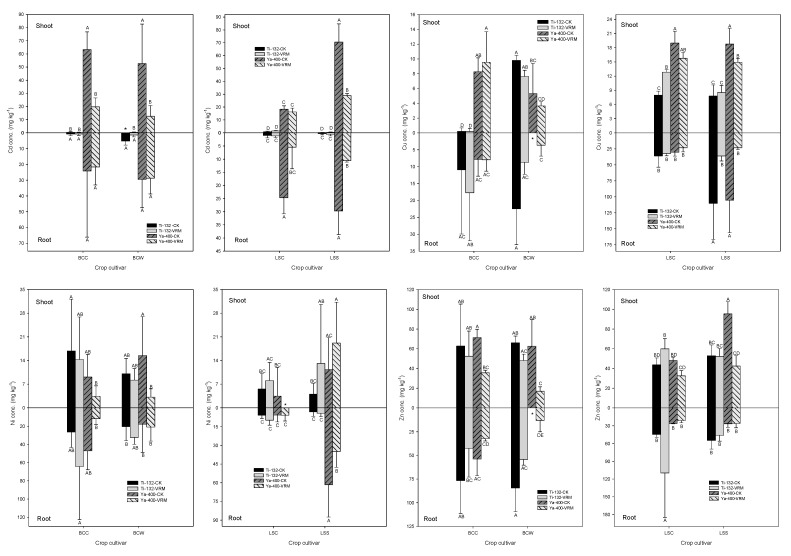
Effects of different treatments on the accumulation of Cd, Cu, Ni, and Zn in the roots and shoots of pak choi and lettuce. The meaning of each code is the same as Table 1. Different letters indicate significant differences across treatments, as determined by one-way ANOVA analysis (LSD test, *p* < 0.05, *n* = 3). * Not detectable.

There was no significant influence of VRM application on the Cd that accumulated in the roots of pak choi compared with CK; however, the Cd accumulation decreased significantly (*p* < 0.05) for the lettuce grown in the Ya-400 soil (Figure 3). The Cd concentrations in the roots of the two lettuce cultivars grown in the Ya-400-CK soil decreased significantly from 25–30 to 5–11 mg kg^−1^. Because of the lower Cd concentration in the Ti-132 soil compared with the Ya-400 soil, the Cd concentrations in the pak choi and lettuce grown in the Ti-132 soil were all less than 15.3 mg kg^−1^. The VRM treatment also significantly decreased the Cd that accumulated in the shoots of leafy vegetables grown in the Ya-400, except for LSC (*p* < 0.05). The Cd concentrations in the shoots of BCC, BCW, and LSS grown in the Ya-400-CK soil were 63.25, 53.62, and 70.53 mg kg^−1^, respectively. There was a 60–75% decrease in the shoots’ Cd concentration under VRM treatment compared with Ya-400-CK. The experimental results also revealed that the Cd accumulation capacity of BCC and LSS was stronger than that of BCW and LSC.

The influence of VRM application on the accumulation of Cu was not similar to that of Cd. Relative to CK, the VRM treatment only significantly decreased the Cu concentrations in the roots and shoots of lettuce (Figure 3). The Cu concentrations in the BCW shoots also decreased compared with CK under the VRM treatment, but the change was not statistically significant. The Cu that accumulated in the roots of LSS significantly decreased from 105–111 to 23–36 mg kg^−1^ under VRM treatment. Additionally, the two lettuce varieties generally accumulated higher concentrations of Cu in the roots and shoots relative to pak choi. Except for BCW shoots and LSS roots grown in the Ya-400 soil, VRM treatment had no significant influence on the accumulation of Ni relative to CK. The VRM application increased the Ni concentration in the roots of two varieties of pak choi and the roots and shoots of the two lettuce cultivars grown in the Ti-132 soil, but the difference was not statistically significant (Figure 3). A maximum Ni concentration of 64 mg kg^−1^ was observed in the roots of BCC grown in Ti-132-VRM. VRM application significantly decreased the Ni concentration in the roots of LSS grown in the Ya-400 soil from 61 to 35 mg kg^−1^. The Ni concentration in the BCW shoots also significantly decreased from 15 to 3 mg kg^−1^ (*p* < 0.05). Except for the LSC grown in the Ti-132 soil, the VRM application decreased the Zn concentration in the roots and shoots of pak choi and lettuce compared with CK (Figure 3). Without VRM application, the Zn concentration in the shoots of the two pak choi varieties grown in the Ti-132 and Ya-400 soils was at levels of 62–72 mg kg^−1^, and the concentrations decreased to 16–53 mg kg^−1^ under VRM amendment. The VRM treatment inhibited the accumulation of Zn in the shoots of the four leafy vegetables, except for LSC and especially for pak choi. The Zn concentrations in the shoots of the four leafy vegetables ranged from 43 to 96 mg kg^−1^ without VRM amendment; under VRM treatment, the concentrations decreased to 16–60 mg kg^−1^.

The experimental results revealed that VRM’s effect on the accumulation of TMs was determined by the species of vegetable. Of the four leafy vegetables, LSS and BCC had higher accumulation capacities than LSC and BCW, respectively. Additionally, the type of TM was also an important factor determining the accumulation of TMs in the vegetables under VRM treatment. VRM application was generally able to decrease the accumulation of the four TMs in the roots and shoots of the two lettuce cultivars, especially Cd, which was the only nonessential TM among the four TMs analyzed. However, for the Cu, Ni, and Zn concentrations in the pak choi, the VRM amendment increased their accumulation under some treatments. The experimental results of this study were similar to those reported by Salati et al. [59], who revealed that the accumulation of Cu, Ni, and Zn in corn increased under organic waste treatment, but the accumulation of Cd decreased. The humus and soluble OM released from the degradation of applied OM possibly chelated with TMs and raised the availability of TMs and thus, their accumulation in the plants [25,58].

Relative to CK, the VRM treatment was able to decrease the accumulation of the four TMs in the roots and shoots of the two lettuce cultivars used in this study overall. The OM in the VRM played an important role in these results. Functional groups, such as amino groups (–NH_2_), hydroxyl groups (–OH), and carbonyl groups (–COOH), of OM can form complexes with TMs and thus, decrease the availability of TMs and their uptake by plants [27,29,60,61,62]. Similar results were reported by previous studies [63,64], which revealed that the application of organic amendment could decrease the availability of Cd and thus, its accumulation in potato and radish plants (*Raphanus sativus* L.). Compared with biochar, the concentrations of TMs in the roots, stems, leaves, and grains of paddy rice (*Oryza sativa* L.) amended with VRM decreased [24]. Besides OM, the increase in essential elements after applying VRM also had a significant effect by decreasing the accumulation of TMs; for instance, phosphate can form complexes with Cd and Zn and decrease their availability [65] and thus, their accumulation in plants [66,67]. An effect of P on decreasing the accumulation of Cd and Zn was also observed in this study. Before the application of VRM, the concentration of available P was at levels of 19–22 and 4.8–9.6 mg kg^−1^ for the Ti-132 and Ya-400 soils, respectively; however, it significantly increased (*p* < 0.05) to 59–97 and 24–55 mg kg^−1^ after VRM application (Table 2 and Table 3).

Similar to the findings of Lam and Lai [18], Cd had the highest BCF of the four TMs in leafy vegetables grown in the Ti-132 and Ya-400 soils, which were at levels of less than 0.5 and 1.0–6.6, respectively (Table 3). These results revealed that, for the nonessential TM (Cd), the BCF increased with increases in soil Cd concentration. Except for lettuce grown in the Ti-132 soil, which was slightly contaminated with Cd, VRM treatment was generally able to decrease the BCF of Cd; however, compared with CK, the BCF of Cd in lettuce grown in the Ti-132 soil increased 1.6–2.6 times under VRM treatment. Similar to the results for BCF, all leafy vegetables grown in the Ya-400 soil had a higher TF for TMs than in the Ti-132 soil, except for Ni (Table 3). Additionally, the BCF of Cd in the Ya-400 soil was higher than for the other three TMs. VRM’s effect on the TF for different crops and treatments was not identical; relative to CK, the TF of Cd and Cu in lettuce grown in two contaminated soils under VRM treatment increased 1.1–4.0 times. For the nonessential TM (Cd), VRM application was able to decrease the BCF and TF of pak choi grown in the Ya-400 soil; however, for lettuce grown in the Ya-400 soil, the TF increased under VRM treatment compared with CK, although the BCF decreased. This phenomenon indicates that more attention should be paid to the management of lettuce grown under Ya-400 conditions with higher concentrations of Cd than under Ti-132 conditions.

### 3.4. Chemical Forms

**Figure 4:**
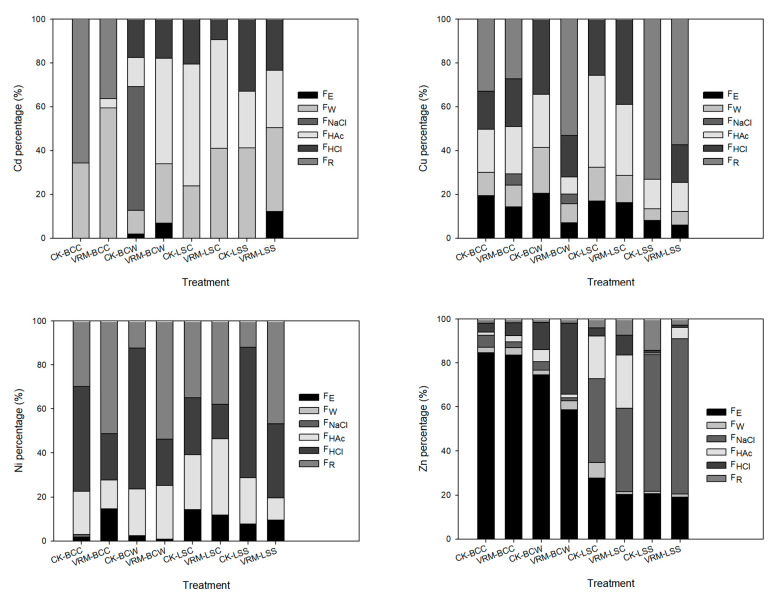
Effects of different treatments on the chemical forms of Cd, Cu, Ni, and Zn in the blanched shoots of pak choi and lettuce. The meaning of each code is the same as in Table 1.

According to previous studies, F_E_ and F_W_ have higher mobility than other chemical forms and are easier to leach out during cooking [15,18]. The experimental result showed that blanching using 90 °C hot water for 90 s was able to decrease the Cd, Cu, Ni, and Zn concentrations compartmentalized in the F_E_ and F_W_ chemical forms of the four leafy vegetables (detailed data not shown). Relative to raw tissues, the decrease in the sum of the F_E_ and F_W_ chemical form concentrations in the four leafy vegetables grown in the two contaminated soils reached 17–92%, 66–100%, 58–100%, and 3–76% for Cd, Cu, Ni, and Zn, respectively; however, higher proportions of the Cd and Zn in the blanched tissues were still compartmentalized in the F_E_ and F_W_ chemical forms compared with Cu and Ni for most of the treatments (Figure 4). The VRM application decreased the sum of the percentages of F_E_ and F_W_ chemical forms of Cd, Cu, and Zn in blanched tissues of pak choi and lettuce grown in the Ya-400 soil, which had higher concentrations of Cd and Ni than the Ti-132 soil; however, the VRM treatment increased the sum of the percentage of F_E_ and F_W_ chemical forms of Cd in the four crops grown in the Ti-132 soil.

Approximately 12–42% and 88–92% of the accumulated Cd in the blanched shoots of pak choi and lettuce grown in Ti-132-CK and Ya-400-CK were compartmentalized in the F_E_ and F_W_ chemical forms, respectively. Under VRM treatment, the above proportions increased to 34–60% and decreased to 55–92% for Ti-132 and Ya-400, respectively. As with previous studies [68,69], the experimental results revealed that the plant species, TM type, and soil type all affected the distribution of TMs’ chemical forms. Of the two soils used in this study, the Ya-400 soil was contaminated with Cd more seriously than the Ti-132 soil, and the accumulated Cd in the shoots of all the leafy vegetables grown in the Ya-400 soil was also higher (Figure 3). VRM treatment could only decrease the proportion of Cd accumulated in the F_E_ and F_W_ chemical forms for pak choi and lettuce grown in the Ya-400 soil, and these forms are more easily taken up by the human digestion system. Approximately 62–88% of accumulated Zn in the blanched shoots of pak choi grown in the Ti-132 and Ya-400 soils was compartmentalized in the F_E_ and F_W_ chemical forms; however, less than 36% of the Zn occurred in these two chemical forms in the blanched shoots of lettuce, regardless of the treatment. Although the Ti-132 soil had a higher Zn concentration than the Ya-400 soil, the four leafy vegetables accumulated similar concentrations of Zn in the shoots. VRM application efficiently decreased Zn concentration and compartmentalization in the F_E_ and F_W_ chemical forms. The experimental results showed that higher proportions of Cd and Zn in the raw and blanched tissues of the four leafy vegetables were in chemical forms with high mobility compared with Cu and Ni. More attention should be paid to these two TMs, especially regarding the only nonessential TM (Cd).

### 3.5. Risk Assessment

Food was the dominant source of TM exposure in humans and accounted for approximately 90% of the intake [70]; moreover, approximately 26% came from vegetables across all foods [71], meaning that the TDI_v_ of Cd, Cu, Ni, and Zn were thus determined to be 0.084, 117, 0.655, and 70.2 μg kg^−1^-BW day^−1^, respectively.

Pak choi and lettuce should be cooked before being eaten, and blanching is the most common way of cooking leafy vegetables in Taiwan. Some TMs in the raw tissues of such vegetables may leach out during cooking, thus decreasing TM concentrations accordingly. Based on Lam et al.’s findings [18], approximately 45–68% and 51–70% of the accumulated Cd and Ni in water spinach leached into boiling water during blanching, respectively. The experimental results of this study showed that approximately 17–71% and 66–92% of the Cd in the F_E_ and F_W_ chemical forms of blanched leafy vegetables leached out compared with raw tissues; therefore, the HQ_v_ calculation in this study was based on the total concentrations of the four TMs and the water content of the edible parts of the four leafy vegetables, and it was assumed that 50% of the accumulated TM would leach out during blanching.

Except for BCW-CK, BCW-VRM, LSC-CK, and LSS-CK grown in the Ti-132 soil, the total HQ_v_ values all exceeded one, which may be considered as hazardous for human health. The four leafy vegetables grown in the Ya-400 soil had higher total HQ_v_ values than those grown in Ti-132 soil because they had higher concentrations of Cd and Ni; moreover, the TDI_v_ values for these two TMs were lower than Cu and Zn. Of the four TMs, the accumulated Cd and Ni in the four leafy vegetables grown in the Ya-400 and Ti-132 soils constituted more than 95% and 42–93% of the total risk through oral intake, respectively (Table 4). The VRM application significantly decreased the HQ_v_ value for Cd for all the leafy vegetables grown in the Ya-400 soil, and the HQ_v_ value for Cd for leafy vegetables grown in the Ya-400 soil decreased from 8.6–34 to levels of 6.8–16. This phenomenon is helpful for risk management because Cd is the only nonessential TM for plant growth. According to the results of the HQ_v_ calculation, however, the oral intake of these leafy vegetables posed a high health risk, except for BCW-CK, BCW-VRM, LSC-CK, and LSS-CK grown in the Ti-132 soil.

## 4. Conclusions

The accumulation of TMs in the four leafy vegetables used in this study differed considerably, even when they were grown in the same TM-contaminated soil. VRM amendment was able to increase soil fertility and thus, observably promoted the growth of pak choi and lettuce; however, accumulations of Cu, Ni, and Zn in the edible parts of the vegetables also increased under VRM treatment. Fortunately, the above three TMs are essential for the growth of plants; the only nonessential TM (i.e., Cd) posed the greatest health risk through oral intake. VRM amendment could significantly decrease Cd accumulation in the edible parts of plants, especially those grown in the Ya-400 soil, which was contaminated with Cd more seriously than the Ti-132 soil. Furthermore, blanching could leach out 17–92% of the Cd compartmentalized in the F_E_ and F_W_ chemical forms with high bioaccessibility. Despite this, planting leafy vegetables in soil contaminated with high concentrations of TMs is prohibited due to unacceptable health risks. Farmers should select crops with low TM accumulation capacity combined with the application of suitable amendments to decrease the risks of oral TM intake.

## Figures and Tables

**Table 1:** The soil properties of Ti-132 under different treatments.

Crop and Treatment ^1^		pH (*w/v* = 1/5)	EC	OM	Avail N	Avail P	Exch Ca	Exch Mg	Exch K
			dS m^−1^	%	mg kg^−1^	mg kg^−1^	mg kg^−1^	mg kg^−1^	mg kg^−1^
BCC	CK	8.55 ± 0.02 A ^2^	0.10 ± 0.01 B	1.59 ± 0.09 D	2.25 ± 3.89 A	19.77 ± 0.21 D	4463.87 ± 51.38 AC	188.42 ± 15.99 C	142.46 ± 20.89 D
	VRM	8.23 ± 0.05 DE	0.11 ± 0.01 AB	2.87 ± 0.26 B	6.74 ± 6.74 A	89.00 ± 5.96 B	4362.40 ± 15.27 BD	368.68 ± 15.93 A	612.03 ± 53.35 B
BCW	CK	8.49 ± 0.06 AB	0.13 ± 0.02 AB	1.50 ± 0.03 D	4.49 ± 3.89 A	20.48 ± 0.18 D	4671.46 ± 183.82 A	202.17 ± 19.40 C	168.04 ± 5.88 D
	VRM	8.02 ± 0.12 F	0.15 ± 0.06 A	3.30 ± 0.43 A	8.95 ± 10.25 A	96.93 ± 8.38 A	4157.62 ± 76.85 D	394.32 ± 46.48 A	826.88 ± 61.36 A
LSC	CK	8.37 ± 0.09 CD	0.13 ± 0.00 AB	1.66 ± 0.04 D	8.99 ± 7.79 A	20.17 ± 1.92 D	4493.56 ± 41.67 AB	187.68 ± 23.60 C	140.02 ± 7.52 D
	VRM	8.13 ± 0.07 EF	0.11 ± 0.00 AB	1.81 ± 0.10 D	8.99 ± 3.91 A	59.25 ± 1.81 C	4249.37 ± 102.52 CD	300.57 ± 10.60 B	365.66 ± 17.58 C
LSS	CK	8.38 ± 0.05 BC	0.12 ± 0.01 AB	1.52 ± 0.04 D	4.47 ± 3.87 A	21.92 ± 1.06 D	4313.42 ± 126.94 BD	197.49 ± 8.59 C	149.51 ± 20.33 D
	VRM	8.29 ± 0.10 CD	0.11 ± 0.00 AB	2.21 ± 0.32 C	6.73 ± 0.02 A	60.30 ± 4.39 C	4269.85 ± 255.57 BD	296.06 ± 19.55 B	346.35 ± 8.89 C

^1^ BCC: *Brassica chinensis* L. var. Chinensis; BCW: *Brassica chinensis* L. cv. wrinkled leaves; LSC: *Lactuca sativa* L. cv. Chinese; LSS: *Lactuca sativa* L. cv. sweet; CK: control; VRM: vermicompost; EC: electrical conductivity; OM: organic matter; Avail: available; Exch: exchangeable. ^2^ Mean ± standard deviation; different letters indicate significant differences across treatments as determined by one-way ANOVA analysis (LSD test, *p* < 0.05, *n* = 3).

**Table 2:** The soil properties of the Y-400 soil under different treatments.

Crop and Treatment ^1^		pH (*w/v* = 1/5)	EC	OM	Avail N	Avail P	Exch Ca	Exch Mg	Exch K
			dS m^−1^	%	mg kg^−1^	mg kg^−1^	mg kg^−1^	mg kg^−1^	mg kg^−1^
BCC	CK	7.18 ± 0.09 C ^2^	0.03 ± 0.00 D	1.48 ± 0.04 D	11.23 ± 3.91 BC	5.62 ± 0.23 DE	2059.09 ± 66.78 A	355.36 ± 15.93 BC	57.78 ± 2.73 E
	VRM	7.65 ± 0.55 A	0.07 ± 0.01 B	2.60 ± 0.55 B	8.98 ± 3.89 BC	49.28 ± 1.58 B	2247.68 ± 88.71 A	484.96 ± 13.35 A	155.75 ± 12.43 B
BCW	CK	7.16 ± 0.06 C	0.04 ± 0.01 D	1.57 ± 0.10 D	8.98 ± 3.87 BC	4.88 ± 0.36 E	1914.34 ± 46.21 A	327.96 ± 7.82 C	65.62 ± 9.06 DE
	VRM	7.54 ± 0.05 AB	0.10 ± 0.00 A	3.07 ± 0.35 A	13.45 ± 0.03 BC	54.83 ± 4.85 A	2385.32 ± 101.12 A	517.24 ± 20.74 A	176.74 ± 13.93 A
LSC	CK	7.17 ± 0.06 C	0.05 ± 0.01 C	1.50 ± 0.10 D	22.43 ± 3.87 A	4.88 ± 0.40 E	2436.52 ± 941.56 A	355.85 ± 11.64 BC	61.57 ± 2.98 E
	VRM	7.26 ± 0.03 BC	0.07 ± 0.01 BC	2.25 ± 0.07 BC	11.22 ± 3.91 BC	25.59 ± 2.01 C	2011.13 ± 312.84 A	393.16 ± 79.67 BC	76.60 ± 7.02 CD
LSS	CK	7.19 ± 0.04 C	0.04 ± 0.00 D	1.38 ± 0.05 D	6.72 ± 6.72 C	9.54 ± 4.72 D	1992.70 ± 1222.34 A	336.26 ± 25.15 C	63.85 ± 3.80 DE
	VRM	7.21 ± 0.07 BC	0.07 ± 0.01 BC	2.01 ± 0.17 C	13.47 ± 0.03 B	24.52 ± 0.50 C	2246.69 ± 66.84 A	406.15 ± 59.01 B	84.93 ± 6.65 C

^1^ The meaning of each code is the same as Table 1. ^2^ Mean ± standard deviation; different letters indicate significant differences across treatments as determined by one-way ANOVA analysis (LSD test, *p* < 0.05, *n* = 3).

**Table 3:** The bioconcentration factors (BCFs) and translocation factors (TFs) of Cd, Cu, Ni, and Zn for pak choi and lettuce grown in Ti-132 and Ya-400 soil under different treatments.

Treatment ^1^		Bioconcentration Factor (BCF)				Translocation Factor (TF)			
		Cd	Cu	Ni	Zn	Cd	Cu	Ni	Zn
Ti-132									
BCC	CK	0.11	0.003	0.32	0.19	0.51	0.02	0.64	0.82
	VRM	0.05	0.002	0.28	0.16	0.26	0.01	0.22	1.23
BCW	CK	NA ^2^	0.08	0.19	0.25	NA	0.44	0.50	0.78
	VRM	0.08	0.11	0.16	0.15	0.19	0.88	0.25	0.89
LSC	CK	0.24	0.10	0.11	0.14	0.82	0.22	0.93	0.98
	VRM	0.41	0.14	0.15	0.20	1.24	0.40	0.82	0.55
LSS	CK	0.04	0.10	0.08	0.16	0.41	0.07	1.24	0.97
	VRM	0.09	0.07	0.24	0.16	0.59	0.24	2.97	1.13
Ya-400									
BCC	CK	5.57	0.40	0.06	0.66	2.62	1.05	0.19	1.32
	VRM	1.66	0.47	0.02	0.32	0.91	1.20	0.29	1.10
BCW	CK	4.70	0.26	0.10	0.53	1.78	NA	0.87	NA
	VRM	1.01	0.17	0.02	0.17	0.44	1.00	0.15	1.28
LSC	CK	1.64	0.90	0.02	0.48	0.74	0.63	0.59	1.82
	VRM	1.45	0.75	NA	0.34	2.96	0.68	NA	1.53
LSS	CK	6.56	0.91	0.07	0.81	2.37	0.18	0.18	3.59
	VRM	2.62	0.71	0.12	0.42	2.73	0.64	0.55	1.61

^1^ The meaning of each code is the same as in Table 1. ^2^ NA: not available because the concentrations in the roots or shoots were not detectable.

**Table 4:** The hazard quotients (HQ_v_) of Cd, Cu, Ni, and Zn for the oral intake of pak choi and lettuce grown under different treatments.

Treatment ^1^		Hazard Quotient (HQ_v_)			
		Cd	Cu	Ni	Zn
Ti-132					
BCC	CK	0.20	0.00	1.05	0.05
	VRM	0.10	0.00	1.00	0.04
BCW	CK	0.00	0.01	0.85	0.06
	VRM	0.13	0.00	0.57	0.03
LSC	CK	0.43	0.00	0.40	0.03
	VRM	0.73	0.01	0.57	0.04
LSS	CK	0.07	0.00	0.31	0.04
	VRM	0.17	0.00	0.99	0.04
Ya-400					
BCC	CK	23.8	0.00	0.57	0.03
	VRM	10.1	0.00	0.24	0.03
BCW	CK	25.7	0.00	1.12	0.04
	VRM	6.84	0.00	0.24	0.01
LSC	CK	8.67	0.01	0.26	0.03
	VRM	8.36	0.01	0.00	0.02
LSS	CK	33.3	0.01	0.78	0.06
	VRM	15.6	0.01	1.45	0.03

^1^ The meaning of each code is the same as in Table 1.

## Data Availability

Not applicable.
